# First Case of the Cervical Lymph Node as the Only Site of Metastasis from Anal Cancer

**DOI:** 10.7759/cureus.1291

**Published:** 2017-05-30

**Authors:** Bo Wang, Sunny Jaiswal, Muhammad W Saif

**Affiliations:** 1 Internal Medicine, Tufts Medical Center; 2 Department of Radiology, Tufts Medical Center; 3 Hematology/Oncology, Tufts Medical Center

**Keywords:** anal cancer, human papilloma virus (hpv), anal squamous cell carcinoma, metastatic anal cancer, metastatic disease, cervical lymph nodes, chemoradiation

## Abstract

Anal squamous cell carcinoma was a previously uncommon malignancy that has steadily increased in incidence with the increased prevalence of human papillomavirus (HPV) and human immunodeficiency virus (HIV). Anal squamous cell carcinoma is typically characterized by local and regional involvement and distant metastases are far less common. Here, we report a case of a 36-year-old female initially diagnosed with anal squamous cell carcinoma manifesting as an anal mass along with an enlarged inguinal lymph node. After receiving chemoradiation therapy, she remained disease-free until recently, when she presented with an isolated left infraclavicular lymph node found on physical examination followed by a biopsy that was consistent with recurrent anal squamous cell carcinoma. The positron emission tomography–computed tomography (PET-CT) uptake of her original left inguinal lymph node was decreased, suggesting improved regional disease, and no other metastases were found. Our case represents a rare occurrence of metastatic anal squamous cell carcinoma to an isolated distal lymph node and reminds physicians not to forget a unusual site of metastasis and prevent any delay in treatment.

## Introduction

Anal cancer accounts for about 2% of all gastrointestinal (GI) cancers and is represented largely by local-regional disease with low potential (15%) for distant metastasis [1]. Squamous cell carcinomas comprise the vast majority of anal cancers. The disease typically demonstrates reasonable five-year survival rates near 80% with cure rates upwards of 60% using chemoradiation [2], with decreased cure rates associated with larger size primary tumors. The cascade of carcinogenesis is marked by initial high-grade intraepithelial neoplasia progressing into malignant transformation [3].

The most common tissue sites for metastasis of squamous cell anal carcinoma include the inguinal lymph nodes and para-aortic lymph nodes, followed by the liver being the most common visceral organ, and the lungs and skin. Distant metastases present rather late in the disease course and rarely occur without existing localized tissue involvement. Metastatic anal cancer, unfortunately, bears a poor prognosis, with an overall five-year survival rate of less than 20%. Treatment for metastatic disease is typically a combination 5-fluouracil (5-FU) with cisplatin as the first-line therapy with a median survival of 12 months [4].

On the other hand, local lymph node involvement is typically present in 30%-40% of patients at diagnosis. Given that the disease typically spreads to local tissue and regional lymph nodes, and uncommonly to distal tissues, anal squamous cell cancer has almost never been reported to spread to an isolated distal lymph node. In this case report, we highlight a rare case of a squamous cell carcinoma that spread to a distal lymph node in the absence of substantial local-regional disease and without evidence of distal metastasis in any other tissue. 

## Case presentation

The patient presented is a 36-year-old female with a history of B-cell acute lymphocytic lymphoma (ALL) diagnosed at age 19, treated with chemotherapy alone in accordance with the ECOG 2993 (Stem Cell Transplantation Compared With Standard Chemotherapy in Treating Patients With Acute Lymphoblastic Leukemia in First Remission) protocol, and since in long-standing remission. She presented to the clinic with an enlarged left-sided inguinal lymph node. A review of systems was unrevealing for any symptoms aside from the palpable node. The lymph node underwent biopsy and was revealed to be positive for pankeratin, cytokeratin 7 (CK7), and transformation-related protein (p63) consistent with squamous cell carcinoma. She was discovered to have a coincident anal lesion on her posterior anal verge, and a biopsy of this lesion demonstrated poorly differentiated squamous cell carcinoma. An in situ hybridization stain for high-risk human papillomavirus (HPV) was weakly positive. Chemoradiation with mytomycin (12 mg/m²) and 5-FU (1000 mg/m²) was initiated, with a second dose four weeks later, and she received concurrent local radiation (50 centigray) to the perianal area and inguinal lymph nodes.

**Figure 1:**
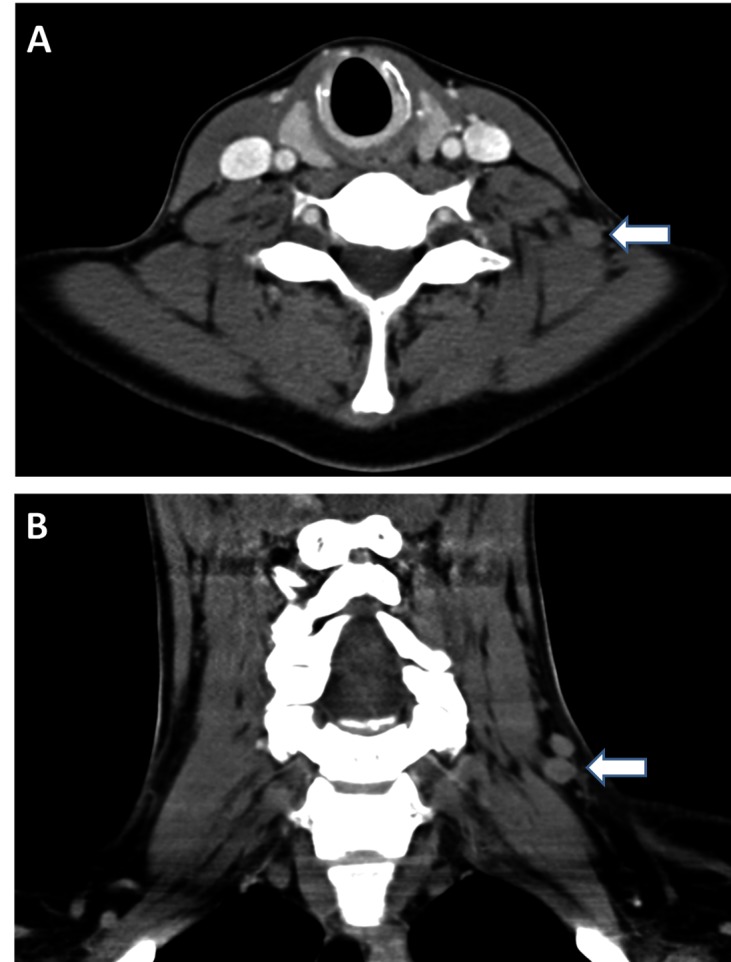
A soft tissue CT scan of the neck indicated lymphadenopathy A) Axial image at the level of the lower neck demonstrating a mildly enlarged left level 5B lymph node measuring up to 0.8 cm in the short axis. B) Coronal image at the level of the lower neck demonstrating two mildly enlarged left level 5B lymph nodes measuring up to 0.8 cm in the short axis.

Eight months following completion of chemoradiation, physical examination revealed enlarged left cervical lymph nodes. A computed tomography (CT) scan of the chest revealed a lower left internal jugular vein thrombus. The patient was started on therapeutic enoxaparin and returned to the clinic one month later with a palpable left-side neck mass. A soft tissue CT scan of the neck indicated lymphadenopathy (Figure 1) and fine needle aspiration followed by excisional biopsy demonstrated squamous cell carcinoma that was morphologically comparable with her prior anal cancer; an in situ hybridization stain for high-risk HPV was weakly positive. A positron emission tomography (PET) and computed tomography (PET-CT) scan one month later showed new left level 5 left infraclavicular lymph nodes with decreased uptake in the original left inguinal node.

She received three cycles of 5-FU and cisplatin with radiation (25 gray). She went on to complete a course of capecitabine (1000 mg/m^2^ twice daily, two weeks on, one week off) and cisplatin (60 mg/m^2^) every three weeks. Secondary to severe thrombocytopenia and neutropenia, cisplatin was withheld in cycles 2, 3, and 6 and she received pegfilgrastim. Scans showed response to radiation and due to elevated risk, she was placed on adjuvant capecitabine for an additional four cycles. Unfortunately, a CT scan revealed new mediastinal adenopathy. Pathology from mediastinoscopy showed metastatic squamous cell carcinoma, consistent with disease progression. She transferred to another facility for a clinical trial and follow-up was lost.

## Discussion

Our patient presents a case of isolated cervical lymph node metastasis as the sole presentation of metastasis in advanced stage anal squamous cell carcinoma in a young female. This case is unique because it represents a rare manifestation and site of metastasis without any visceral or local disease, and no such case has been reported to the best of our knowledge in our review of the medical literature. Cancers of the anal region may spread based on lymphatic drainage – drainage to the internal iliac lymph nodes is typical for lesions above the pectinate line, while drainage to the superficial inguinal lymph nodes is typical for lesions below the pectinate line. Iliac lymph nodes eventually drain to the paraaortic nodes.

The initial local-regional involvement of the inguinal lymph node was appropriate based on the above lymphatic drainage. However, there is no likely explanation for the lymphatic spread of her anal cancer to the cervical lymph node. Therefore, she more likely suffered from the hematogenous spread of her anal cancer. Hematogenous spread is rare in anal cancer and occurs in less than 10% of cases [5]. Like cancers of the colorectal region, the most common organ metastasis is to the liver. Other rare cases of distant metastases have been reported to the brain [6], bone marrow [7], and iris [8]. Hematogenous spread to the cervical lymph node as in this case represents a very rare and unlikely case of metastatic anal cancer.

Treatment of anal cancer is based on staging and may involve radiation therapy, chemotherapy, and surgery. Local disease and locally advanced disease are managed with a combination of chemotherapy, radiation, and surgery, while metastatic disease is managed with chemoradiation therapy or chemotherapy alone. Over time, radiation therapy with concurrent infusion chemotherapy involving 5-FU and mytomycin has evolved into the standard care for squamous cell anal cancer [9].  For metastatic disease, surgery is at times indicated for recurrent or residual disease.

About 10%-20% of patients experience distant relapsed disease. Prognosis is relatively poor with only 10% of patients demonstrating a greater than two-year survival rate. However, treatment data is limited for metastatic disease as metastatic anal cancer is less common. ESMO guidelines suggest considering surgery for persistent or progressive disease in the inguinal lymph nodes [10]. In this case, the patient's inguinal node diminished in size with chemoradiation, yet she developed new metastatic disease in a distant lymph node in the cervical area. Due to limited trial data, it remains unknown whether there are clear benefits to surgical removal of isolated metastases for squamous cell anal cancer. 

## Conclusions

In anal cancer, local lymph node involvement is typically seen in 30%-40% of patients at diagnosis; however, metastasis to the internal iliac lymph nodes is typical for lesions above the pectinate line, while drainage to the superficial inguinal lymph nodes is typical for lesions below the pectinate line, and iliac lymph nodes eventually drain to the paraaortic nodes.  Distant metastases occur in some patients, but the liver is the most common site. Given that disease typically spreads to local tissue and regional lymph nodes, and uncommonly to distal tissues, our rare case showed a spread of anal squamous cell carcinoma to a distal lymph node in the absence of substantial local-regional disease and without evidence of distal metastasis in any other tissue. Data is lacking to answer the potential role of surgery, such as metastasectomy, in the management of metastatic anal carcinoma. Such an occurrence as this is rare in anal cancer and presents a management dilemma for a multi-disciplinary team.
